# Subpleural fibrotic interstitial lung abnormalities are implicated in non-small cell lung cancer radiotherapy outcomes

**DOI:** 10.2478/raon-2023-0018

**Published:** 2023-04-20

**Authors:** Makoto Ito, Takuma Katano, Hiroaki Okada, Ami Sakuragi, Yoshitaka Minami, Souichiro Abe, Sou Adachi, Yukihiko Oshima, Wataru Ohashi, Akihito Kubo, Takayuki Fukui, Satoru Ito, Kojiro Suzuki

**Affiliations:** Department of Radiology, Aichi Medical University, Aichi, Japan; Department of Respiratory Medicine and Allergology, Aichi Medical University, Aichi, Japan; Department of Central Radiology, Aichi Medical University, Aichi, Japan; Department of Biostatistics, Clinical Research Center, Aichi Medical University, Aichi, Japan; Division of Chest Surgery, Department of Surgery, Aichi Medical University, Aichi, Japan

**Keywords:** interstitial lung abnormality, subpleural fibrosis, non-small cell lung cancer, radiotherapy, radiation pneumonitis, survival analysis

## Abstract

**Background:**

The relationship between interstitial lung abnormalities (ILAs) and the outcomes of lung cancer radiotherapy is unclear. This study investigated whether specific ILA subtypes are risk factors for radiation pneumonitis (RP).

**Patients and methods:**

This retrospective study analysed patients with non-small cell lung cancer treated with radical-intent or salvage radiotherapy. Patients were categorised into normal (no abnormalities), ILA, and interstitial lung disease (ILD) groups. The ILA group was further subclassified into non-subpleural (NS), subpleural non-fibrotic (SNF), and subpleural fibrotic (SF) types. The Kaplan–Meier and Cox regression methods were used to determine RP and survival rates and compare these outcomes between groups, respectively.

**Results:**

Overall, 175 patients (normal, n = 105; ILA-NS, n = 5; ILA-SNF, n = 28; ILA-SF, n = 31; ILD, n = 6) were enrolled. Grade ≥2 RP was observed in 71 (41%) patients. ILAs (hazard ratio [HR]: 2.33, p = 0.008), intensity-modulated radiotherapy (HR: 0.38, p = 0.03), and lung volume receiving 20 Gy (HR: 54.8, p = 0.03) contributed to the cumulative incidence of RP. Eight patients with grade 5 RP were in the ILA group, seven of whom had ILA-SF. Among radically treated patients, the ILA group had worse 2-year overall survival (OS) than the normal group (35.3% vs 54.6%, p = 0.005). Multivariate analysis revealed that the ILA-SF group contributed to poor OS (HR: 3.07, p =0.02).

**Conclusions:**

ILAs, particularly ILA-SF, may be important risk factors for RP, which can worsen prognosis. These findings may aid in making decisions regarding radiotherapy.

## Introduction

Lung cancer is one of the most common and deadliest cancers worldwide, with non-small cell lung cancer (NSCLC) accounting for approximately 85% of all cases.^1,2^ Radiotherapy is a typical non-surgical curative treatment for localised NSCLC.^3^ Recently, the integration of immunotherapy with chemoradiation has revolutionised the treatment of locally advanced NSCLC. For instance, the PACIFIC trial reported a 5-year overall survival (OS) rate of 42.9% and progression-free survival (PFS) rate of 33.1% among individuals who received treatment with durvalumab, an anti-programmed cell death-ligand 1 antibody, after chemoradiotherapy for stage III NSCLC.^4^ However, radiation pneumonitis (RP) is a major concern with this treatment strategy. Patients with grade ≥ 2 RP (according to the Common Terminology Criteria for Adverse Events [CTCAE]) from prior chemoradiotherapy cannot use durvalumab.^5^ Additionally, RP causes respiratory insufficiency that severely affects the quality of life, leading to poor prognosis and even death.^6^ Risk factors should be identified before treatment to avoid grade 2 or more severe RP.

Various risk factors for RP have been reported, such as age, tumour size, and tumour location.^7^ Interstitial lung disease (ILD) is a significant pre-disposing condition. Radiotherapy for lung cancer complicated by ILD induces a high RP rate and borders on being contraindicated.^8^ In contrast, the influence of interstitial lung abnormalities (ILAs) on RP risk remains unclear. Studies investigating the relationship between ILAs and RP are limited, and their results are inconsistent; additionally, no consensus has been reached.^9,10^ ILAs are specific computed tomography (CT) findings that are potentially compatible with ILD in patients without clinical indication of the disease. ILAs had been originally described as non-dependent abnormalities affecting more than 5% of any lung zone; however, they overlapped with other disease concepts.^11^ In 2020, the Fleischner Society clarified the definition of ILAs and further classified them into three subtypes—namely, non-subpleural (NS), subpleural non-fibrotic (SNF), and subpleural fibrotic (SF).^12^ Nevertheless, there are no reports describing lung cancer radiotherapy's effect on these newly defined ILAs.

Therefore, in this study, we retrospectively reviewed the data of patients with NSCLC who underwent radiotherapy and conducted a survival analysis to investigate how the novel ILA subtypes relate to RP risk.

## Patients and methods

### Patients

We retrospectively reviewed the medical records of patients with NSCLC treated with radiotherapy between January 2010 and November 2021. We included patients treated with radical-intent radiotherapy for locally advanced NSCLC or with salvage radiotherapy for locoregional recurrence postoperatively. Of the 188 consecutive patients who met these criteria, 9 who received stereotactic body radiation therapy for salvage and 4 who had a short follow-up duration (< 6 months) were excluded. We collected information from the remaining 175 patients’ medical records, including age, sex, Eastern Cooperative Oncology Group performance status, smoking history, blood sampling, pulmonary function tests, pathology, and imaging data. Patients treated with radical-intent radiotherapy were staged according to the 8^th^ edition of the Union for International Cancer Control tumour–node–metastasis classification. This study was approved by the Ethics Committee of Aichi Medical University (approval no. 2021-545) with an opt-out approach regarding the analysis before this study. Al l procedures involved in the study adhered to the principles of the Declaration of Helsinki. The re quirement for the acquisition of informed consent from patients was waived owing to the retrospective nature of this study.

### Radiotherapy

Patients were immobilised in the supine position using an external vacuum-type body mould and/or thermoplastic body mask, and a CT scan with a 2-mm slice thickness was conducted for treatment planning. Respiratory motion was confirmed by obtaining CT images in both the expiratory and inspiratory phases or during shallow breathing. Both the target volume and normal organ structures were contoured using treatment planning systems (CMS XiO, Elekta, St Louis, MO, USA; Eclipse, Varian Medical Systems, Palo Alto, CA, USA; or Maestro, MIM Software, Inc., Cleveland, OH, USA). The clinical target volume (CTV) was defined as a 0–5-mm expansion of the primary tumour and metastatic lymph nodes. To account for respiratory migration, a 5–15-mm margin was added to the CTV to define the planning target volume (PTV). Radiotherapy was delivered by 6- or 10-MV x-rays from linear accelerators (Clinac iX or TrueBeam STx, Varian Medical Systems, Palo Alto, CA, USA). Overall, 60 Gy in 30 fractions was delivered to the PTV, and 119 (68%) patients received prophylactic elective nodal irradiation of 40 Gy in 20 fractions to the lymph node area. In exceptional cases, 11 (6%) patients received dose escalation up to 64–70 Gy.

Additionally, six (3%) patients underwent hypofractionated radiotherapy of up to 2.7–3 Gy per fraction to shorten the duration of treatment. The irradiation methods used were three-dimensional conformal or intensity-modulated radiotherapy (IMRT). Two-step IMRT was used for the entire period or only as boost irradiation of 20 Gy in 10 fractions.

The dose constraints were as follows: the goals for the global and spinal maximum doses were < 107% (< 125% allowed) and < 50 Gy (< 52 Gy allowed), respectively, whereas the goals for the lung volumes were 5 Gy (V5) < 60%, V20 < 25%, and mean lung dose < 12 Gy. Vx refers to the percentage of the lung volume receiving > x Gy. The goal for the mean heart dose was < 20 Gy. Allowance values for lung and heart doses were not set; however, every effort was made to reduce them as much as possible.

### Chemotherapy and immunotherapy

Patient age, general condition, and organ function determined the appropriateness of concurrent chemotherapy and its regimen. The most commonly used regimen was weekly carboplatin/paclitaxel, and cisplatin/docetaxel or cisplatin/vinorelbine was also administered. Durvalumab has been available to patients at our institution since October 2018. We performed CT after concurrent chemoradiation therapy was completed to ensure that there was no disease progression or grade ≥ 2 RP before administration. Durvalumab was intravenously administered at a dose of 10 mg/kg every 2 weeks for 1 year. If grade 2 RP appeared, the administration was suspended until the patient recovered to grade 1. We stopped administration in cases of serious adverse events, such as grade ≥ 3 RP or confirmed disease progression.

### ILA classification and treatment outcomes

ILAs were defined according to the Fleischner Society classification.^12^ The grouping was primarily performed by two physicians: a chest physician engaged in ILDs and a diagnostic radiologist engaged in chest radiology. They reviewed medical records including past history, family history, and reasons for imaging studies before reviewing CT images. Next, they used diagnostic chest CT images taken before treatment for grouping. All patients were classified into the following three groups: normal (no abnormalities), ILA, and ILD. The ILA group was further subdivided into NS, SNF, and SF groups. This process was performed independently by a chest physician and a diagnostic radiologist without discussion and was later collated. Grouping of any discrepant cases was finalized by consultation among the two, with another radiologist acting as an intermediary. Details of the CT protocols used for grouping are as follows. SOMATOM Definition AS/AS+/Flash (Siemens Healthcare, München, Germany) and Light Speed VCT VISION (General Electric Healthcare, Milwaukee, Wisconsin, USA) CT scanners were used. Slice thickness was 2–2.5-mm. Tube voltage was 120 kVp. Tube current was auto exposure control. Scan mode was helical acquisition. Pitch factor was 0.8–1.5. Rotation time was 0.3–0.5.

We measured the time to the event from the date of radiotherapy commencement. Toxicity was graded according to the CTCAE version 5.0. Grade 2 RP was considered an event with steroid administration for chest symptoms; administration of general cough suppressants was not included. Survival analysis was performed for patients under radical treatment.

### Statistical analyses

All statistical analyses were performed using EZR version 1.55 (Saitama Medical Center, Jichi Medical University, Saitama, Japan) based on R and R commanders.^13^ Patient characteristics were compared using Fisher's exact test for categorical variables and Student's t-test or the Mann–Whitney U test for continuous variables. Additionally, the Kaplan–Meier method was employed to estimate the cumulative incidence of RP and survival rates, and comparisons were performed using Gray's or log-rank test with post-hoc Bonferroni analyses for multiple comparisons. Finally, a Cox proportional hazards model was used for univariate and multivariate (stepwise elimination) analyses to determine factors contributing to RP and survival rates. Statistical significance was set at *p* < 0.05. Factors demonstrating *p* < 0.2 in the univariate analysis were included in the multivariate analysis. Receiver operating characteristic (ROC) curves were used to evaluate the relationship between RP and lung V20. The cut-off point was determined based on the Youden index, and well-balanced sensitivity and specificity values were obtained.^14^

## Results

### Patient characteristics

The final analysis included 175 patients, with the normal, ILA, and ILD groups comprising 105, 64, and 6 patients, respectively. Table 1 summarises patient characteristics.

**TABLE 1.** Patient characteristics

**Characteristic**	**Normal (*n* = 105)**	**ILA (*n* = 64)**	**ILD (*n* = 6)**	***p***
**Age** (years)	71 (41–90)	72 (60–86)	74 (63–86)	0.01
**Sex** (men)	82 (78%)	52 (81%)	6 (100%)	0.70
**Performance status**	–	–	–	0.42
0	53 (50%)	37 (58%)	4 (66%)	–
1	41 (39%)	19 (30%)	1 (17%)	–
2	7 (7%)	7 (11%)	1 (17%)	–
3	4 (4%)	1 (1%)	0 (0%)	–
**Pack year**	45 (0–171)	47 (0–122)	48.5 (20–84)	0.98
**Pulmonary function test**				
%VC (%)	99.1 (41.8–144.2)	103.0 (41.8–147.5)	86.9 (55.7–98.8)	0.08
FEV1 (L)	1.9 (0.6–3.6)	2.2 (0.9–3.5)	2.0 (1.5–2.1)	0.12
FEV1/FVC (%)	69.2 (34.1–98.1)	69.8 (36.0–85.0)	74.8 (62.2–86.8)	0.36
**KL-6** (U/mL)	282 (151–3957)	370 (172–896)	393 (204–859)	0.65
**Pathology**	–	–	–	0.01
Adenocarcinoma	59 (56%)	21 (33%)	2 (33%)	–
Squamous cell carcinoma	40 (38%)	38 (59%)	4 (67%)	–
Others	6 (6%)	5 (8%)	0 (0%)	–
**Lower-lobe primary lesion** (radical-intent)	19 (22%)	14 (33%)	3 (75%)	0.20
**TNM classification** (radical-intent)				
T classification	–	–	–	0.53
1	13 (15%)	3 (7%)	0 (0%)	–
2	23 (26%)	12 (28%)	2 (50%)	–
3	19 (22%)	13 (30%)	0 (0%)	–
4	32 (37%)	15 (35%)	2 (50%)	–
N classification	–	–	–	0.32
0	7 (9%)	8 (19%)	0 (0%)	–
1	16 (18%)	8 (19%)	1 (25%)	–
2	42 (48%)	16 (37%)	2 (50%)	–
3	22 (25%)	11 (26%)	1 (25%)	–
Stage	–	–	–	0.40
II A	3 (3%)	1 (2%)	0 (0%)	–
II B	5 (6%)	6 (14%)	0 (0%)	–
III A	34 (39%)	15 (35%)	3 (75%)	–
III B	36 (41%)	14 (33%)	0 (0%)	–
III C	9 (11%)	7 (16%)	1 (25%)	–
**Concurrent chemotherapy**	65 (62%)	33 (52%)	2 (33%)	0.20
Carboplatin + paclitaxel	30 (46%)	18 (55%)	2 (100%)	–
Cisplatin + docetaxel	22 (34%)	8 (24%)	0 (0%)	–
Cisplatin + vinorelbine	13 (20%)	7 (21%)	0 (0%)	–
**Durvalumab**	17 (16%)	9 (14%)	0 (0%)	0.83
**PD-L1** (positive)	36 (34%)	23 (36%)	0 (0%)	0.99
**Mutation**	–	–	–	0.99
EGFR	8 (7%)	5 (8%)	1 (17%)	–
ALK	6 (6%)	2 (3%)	1 (17%)	–
None/unknown	91 (87%)	57 (89%)	4 (66%)	–
**Radiotherapy method**	–	–	–	0.08
3DCRT	80 (76%)	40 (63%)	5 (83%)	–
IMRT	25 (24%)	24 (37%)	1 (17%)	–
**ENI**	77 (73%)	38 (59%)	4 (67%)	0.06
**Hypofractionated radiotherapy**	3 (3%)	3 (5%)	0 (0%)	0.67
**Dose escalation**	6 (6%)	5 (8%)	0 (0%)	0.75
**Lung dose**				
V5 (%)	34.5 (4.3–90.8)	40.3 (5.8–95.7)	34.6 (10.6–66.8)	0.18
V10 (%)	27.1 (2.7–67.7)	32.4 (3.7–81.4)	27.8 (8.8–49.9)	0.19
V20 (%)	19.8 (1.1–44.8)	22.0 (1.0–48.0)	22.6 (7.3–33.0)	0.59
Mean (Gy)	10.8 (1.7–23.5)	11.8 (2.0–19.6)	12.3 (3.8–18.0)	0.53
**Median follow-up** (years)	1.8 (0.1–12.1)	1.3 (0.1–9.9)	0.9 (2.1–3.9)	0.06
**Median follow-up: survivors** (years)	2.9 (0.6–12.1)	1.9 (0.6–9.9)	0.9 (0.9–0.9)	0.51

Data are presented as median (range) or number (%). The *p*-values represent comparisons between the normal and ILA groups.

ALK = anaplastic lymphoma kinase; EGFR = epidermal growth factor receptor; ENI = elective nodal irradiation; FEV1 = forced expiratory volume in 1 second; FEV1/FVC = forced expiratory volume % in 1 second; ILAs = interstitial lung abnormalities; ILD = interstitial lung disease; IMRT = intensity-modulated radiotherapy; KL-6 = Krebs von den Lungen-6; *n =* total number of patients; PD-L1 = programmed cell death-ligand 1; TNM = tumour–node–metastasis; VC = vital capacity percentage; Vx = percentage of lung volume receiving > x Gy; 3DCRT = three-dimensional conformal radiotherapy%

**FIGURE 1.**
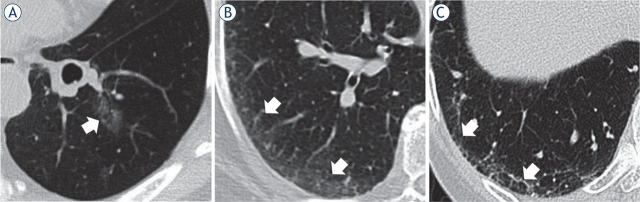
Examples of interstitial lung abnormality subcategories. **(A)** non-subpleural, **(B)** subpleural non-fibrotic, and **(C)** subpleural fibrotic. White arrows point to lesions.

The p-values represent comparisons between the normal and ILA groups. The normal group was younger and had more patients with adenocarcinomas than the ILA group. The median follow-up period of the 70 surviving patients was 2.5 (range, 0.6–12.1) years. The 64 patients with ILA were subcategorised into NS, SNF, and SF subtypes comprising 5, 28, and 31 patients, respectively. Examples of the ILA subcategories are shown in Figure 1. The two specialists (a chest physician and a diagnostic radiologist) matched on 125 of the 175 patient classifications (72%), and the remaining cases were grouped by discussion among the three physicians.

### Radiation pneumonitis

Grade ≥ 2 RP was identified in 71 (41%) patients. Of the 105 patients in the normal group, 21 (20%), 8 (8%), and 1 (1%) exhibited grades 2, 3, and 4, respectively. Of the 64 patients in the ILA group, grades 2, 3, 4, and 5 were observed in 19 (30%), 8 (13%), 2 (3%), and 8 (13%) individuals, respectively. Of the six patients in the ILD group, 2 (33%) and 3 (50%) showed grades 2 and 3, respectively. Seven of the eight patients with grade 5 RP were in the ILA-SF group, and one was in the ILA-SNF group (details shown in Table 2).

**TABLE 2.** Clinical details of eight patients with grade 5 radiation pneumonitis

**Case**	**Age**	**ILA subcategory**	**Radiotherapy method**	**Concurrent chemotherapy**	**Durvalumab**	**Lung V20 (%)**	**Mean lung dose (Gy)**	**Days to death**
1	64	SF	IMRT	Yes	No	19.2	12.8	104
2	73	SF	3DCRT	Yes	No	21.4	13.9	210
3	73	SF	IMRT	No	No	30.6	15.7	123
4	76	SF	IMRT	Yes	Yes	34.1	17.9	155
5	77	SNF	3DCRT	No	No	33.2	16.5	155
6	79	SF	IMRT	Yes	No	31.5	17.3	160
7	79	SF	3DCRT	No	No	19.4	9.7	40
8	80	SF	3DCRT	No	No	27.6	15.2	99

ILAs = interstitial lung abnormalities; IMRT = intensity-modulated radiotherapy; SF = subpleural fibrotic; SNF = subpleural non-fibrotic; V20 = percentage of lung volume receiving > 20 Gy; 3DCRT = three-dimensional conformal radiotherapy

**FIGURE 2.**
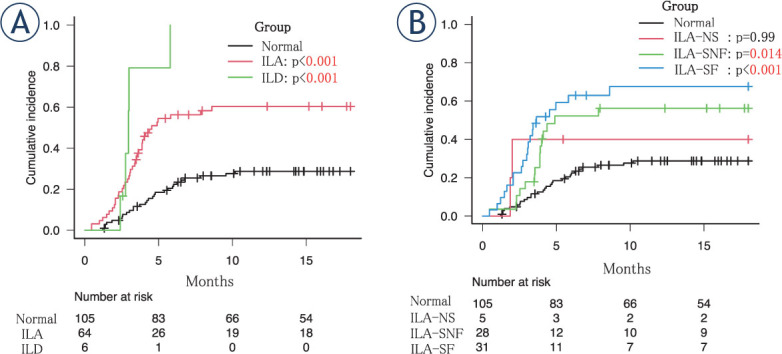
Cumulative incidence of grade ≥ 2 radiation pneumonitis by group **(A)** and subgroup **(B)**. ILAs = interstitial lung abnormalities; ILD = interstitial lung disease; NS = non-subpleural; SF = subpleural fibrotic; SNF = subpleural non-fibrotic

Figure 2 shows the cumulative incidence of grade ≥ 2 RP by group or subgroup. The incidence of RP was significantly higher in the ILA group than in the normal group (p < 0.001), particularly between the SNF (p = 0.014) and SF groups (p < 0.001) and the normal group. Table 3 presents the results of the univariate and multivariate analyses.

**TABLE 3.** Univariate and multivariate analyses of the cumulative incidence of grade ≥ 2 radiation pneumonitis

**Parameter**	**Univariate analysis**		**Multivariate analysis**	

	**HR (95% CI)**	***p***	**HR (95% CI)**	***p***
**ILA**	2.95 (1.81–4.82)	<0.001	2.33 (1.18–4.61)	0.01
**Age** (years)	1.02 (0.99–1.05)	0.14	0.99 (0.95–1.03)	0.78
**Sex** (men)	1.02 (0.57–1.84)	0.94	–	–
**Performance status** (0,1 *vs*. 2,3)	1.17 (0.56–2.44)	0.68	–	–
**Pack year**	1.01 (0.99–1.01)	0.27	–	–
**%VC** (%)	0.99 (0.98–1.01)	0.96	–	–
**FEV1** (L)	1.01 (0.64–1.55)	0.99	–	–
**FEV1/FVC** (%)	0.99 (0.97–1.01)	0.67	–	–
**KL-6** (U/mL)	1.00 (0.99–1.01)	0.09	1.00 (0.99–1.01)	0.13
**Pathology** (adenocarcinoma)	0.58 (0.35–0.96)	0.04	0.85 (0.42–1.72)	0.65
**Lower-lobe primary lesion**	1.22 (0.69–2.12)	0.49	–	–
**T classification** (T4 *vs*. others)	0.81 (0.47–1.39)	0.44	–	–
**N classification** (positive)	1.18 (0.51–2.72)	0.70	–	–
**Concurrent chemotherapy**	1.17 (0.72–1.88)	0.53	–	–
**Durvalumab**	1.32 (0.72–2.40)	0.37	–	–
**PD-L1**	1.11 (0.54–2.25)	0.78	–	–
**Mutation**	1.24 (0.62–2.49)	0.54	–	–
**Radiotherapy method** (IMRT)	0.69 (0.39–1.19)	0.18	0.38 (0.16–0.91)	0.03
**ENI**	1.06 (0.65–1.75)	0.81	–	–
**Lung dose**				
V5 (%)	2.14 (0.67–6.82)	0.19	^*^	^*^
V10 (%)	3.93 (0.85–18.2)	0.08	^*^	^*^
V20 (%)	34.8 (3.05–396.4)	0.004	54.8 (1.52–1977.0)	0.03
Mean (Gy)	1.10 (1.04–1.17)	<0.001	^*^	^*^

CI = confidence interval; ENI = elective nodal irradiation; FEV1 = forced expiratory volume in 1 second; FEV1/FVC = forced expiratory volume % in 1 second; HR = hazard ratio; ILAs = interstitial lung abnormalities; IMRT = intensity-modulated radiotherapy; KL-6 = Krebs von den Lungen-6; N = node; PD-L1 = programmed cell death-ligand 1; RP = radiation pneumonitis; T = tumour; %VC = vital capacity percentage; Vx = percentage of lung volume receiving > x Gy; *vs*. = versus

* Variables with high multicollinearity were excluded.

ILAs (hazard ratio [HR]: 2.33, p = 0.01), IMRT (HR: 0.38, p = 0.03), and lung V20 (HR: 54.8, p = 0.03) were significantly associated with the cumulative incidence of grade ≥ 2 RP in the multivariate analyses.

ROC analysis using V20 showed intermediate grade ≥ 2 RP predictive accuracy in the normal group; the area under the curve (AUC) was 0.72 (95% confidence interval [CI]: 0.60–0.83). The sensitivity and specificity for a cut-off value of 21% for V20 were 76% and 67%, respectively. Conversely, the predictive accuracy in the ILA group was low, with an AUC of 0.59 (95% CI: 0.45–0.75); furthermore, the sensitivity and specificity for a cut-off value of 19% were 76% and 56%, respectively. During follow-up after radiotherapy, five patients received molecular-targeted therapies, and two had grade ≥ 2 RP. However, it was not until 2 years and 10 years after radiotherapy that the two patients, respectively, began receiving molecular-targeted therapies, which had already cured their RP.

### Survival

The 2-year OS and PFS rates for the 134 radically treated patients were 47.8% (95% CI: 38.5–56.4%) and 21.2% (95% CI: 14.4–28.9%), respectively. Patients in the ILA group had worse 2-year OS than those in the normal group (35.3% *vs*. 54.6%, *p* = 0.005); however, the multivariate analysis showed no significant difference. No significant difference was found in the 2-year PFS between the ILA and normal groups (17.3% vs 24.1%, p = 0.26).

The characteristics of the patients treated with radical-intent radiotherapy are summarised in Table 4. The parameters to be entered into the multivariate analysis are listed separately for the normal and ILA-SF groups.

**TABLE 4.** Characteristics of patients treated with radical-intent radiotherapy

**Characteristic**	**Normal (*n* = 87)**	**ILA-SF (*n* = 24)**	***p***
**Age** (years)	69 (41–90)	73 (60–82)	0.09
**Performance status**	–	–	0.39
0	41 (47%)	12 (50%)	–
1	36 (41%)	8 (33%)	–
2	6 (7%)	4 (17%)	–
3	4 (5%)	0 (0%)	–
**Pack year**	45 (0–171)	53 (0–122)	0.38
**KL-6** (U/mL)	294 (151–1607)	399 (208–705)	0.91
**Pathology**	–	–	0.09
Adenocarcinoma	46 (53%)	7 (29%)	–
Squamous cell carcinoma	35 (40%)	14 (58%)	–
Others	6 (7%)	3 (13%)	–
**Lower-lobe primary lesion**	19 (22%)	6 (25%)	0.79
**T classification**	–	–	0.36
1	13 (15%)	1 (4%)	–
2	23 (26%)	10 (42%)	–
3	19 (22%)	5 (21%)	–
4	32 (37%)	8 (33%)	–
**Concurrent chemotherapy**	58 (67%)	13 (54%)	0.33
**Durvalumab**	15 (17%)	1 (4%)	0.19
**Mutation**	8 (13%)	3 (18%)	0.70
**Median follow-up: survivors** (years)	2.8 (0.6–12.1)	1.5 (0.7–6.0)	0.46

Data are presented as median (range) or number (%).

ILA-SF = subpleural fibrotic interstitial lung abnormalities; KL-6 = Krebs von den Lungen-6; *n =* total number of patients

No significant bias was found between the two groups; however, only one patient in the ILA-SF group received durvalumab. Patients in the ILA-SF group had significantly worse 2-year OS (29.2% vs 54.6%, p < 0.001) and PFS (9.7% vs 24.1%, p = 0.009) rates than those in the normal group. Table 5 shows the results of the univariate and multivariate analyses for the OS and PFS rates.

**TABLE 5.** Univariate and multivariate analyses for overall and progression-free survival rates

**Parameter**	**Overall survival**				**Progression-free survival**			

	**Univariate**		**Multivariate**		**Univariate**		**Multivariate**	

	**HR (95% CI)**	***P***	**HR (95% CI)**	***p***	**HR (95% CI)**	***p***	**HR (95% CI)**	***p***
**ILA-SF**	2.59 (1.51–4.43)	<0.001	3.07 (1.17–8.10)	0.02	1.88 (1.16–3.05)	0.01	1.95 (0.91–4.14)	0.08
**Age** (years)	1.02 (0.99–1.04)	0.09	0.95 (0.91–0.99)	0.04	1.01 (0.98–1.03)	0.59	–	–
**Sex** (men)	1.21 (0.70–2.10)	0.49	–	–	0.91 (0.58–1.45)	0.71	–	–
**Performance status** (0,1 *vs*. 2,3)	1.90 (1.07–3.39)	0.03	0.82 (0.19–3.38)	0.78	2.81 (1.66–4.78)	<0.001	1.35 (0.46–3.93)	0.59
**Pack year**	1.01 (1.00–1.01)	0.04	1.01 (0.99–1.02)	0.14	1.00 (0.99–1.01)	0.67	–	–
**%VC** (%)	0.99 (0.98–1.01)	0.56	–	–	0.99 (0.98–1.01)	0.51	–	–
**FEV1** (L)	0.84 (0.55–1.29)	0.43	–	–	0.92 (0.62–1.36)	0.68	–	–
**FEV1/FVC** (%)	0.99 (0.97–1.02)	0.92	–	–	1.01 (0.98–1.02)	0.75	–	–
**KL-6** (U/mL)	1.00 (0.99–1.01)	0.11	1.00 (0.99–1.01)	0.34	1.01 (1.00–1.02)	0.03	1.00 (0.99–1.01)	0.40
**Pathology** (adenocarcinoma)	0.46 (0.31–0.69)	<0.001	0.18 (0.08–0.44)	<0.001	0.54 (0.36–0.79)	0.002	0.34 (0.18–0.65)	0.001
**Lower-lobe primary lesion**	1.60 (1.02–2.53)	0.04	1.03 (0.39–2.68)	0.95	1.09 (0.72–1.65)	0.69	–	–
**T classification** (T4 *vs*. others)	1.15 (0.74–1.79)	0.53	–	–	1.47 (0.99–2.17)	0.05	1.53 (0.78–2.99)	0.21
**N classification** (positive)	1.02 (0.49–2.12)	0.95	–	–	0.88 (0.48–1.60)	0.67	–	–
**Concurrent chemotherapy**	0.51 (0.33–0.80)	0.004	0.19 (0.07–0.56)	0.002	0.60 (0.41–0.89)	0.01	0.65 (0.31–1.30)	0.22
**Durvalumab**	0.22 (0.08–0.60)	0.003	0.43 (0.11–1.69)	0.22	0.34 (0.18–0.65)	0.001	0.40 (0.17–0.93)	0.03
**PD-L1** (positive)	0.77 (0.39–1.50)	0.44	–	–	1.03 (0.59–1.79)	0.92	–	–
**Mutation**	0.76 (0.47–1.22)	0.26	–	–	0.96 (0.64–1.42)	0.83	–	–
**Radiotherapy method** (IMRT)	0.75 (0.43–1.30)	0.31	–	–	0.86 (0.55–1.34)	0.50	–	–
**ENI**	0.83 (0.49–1.38)	0.47	–	–	0.99 (0.64–1.56)	0.99	–	–
**Lung dose**								
V5 (%)	0.45 (0.12–1.72)	0.24	–	–	0.56 (0.19–1.66)	0.30	–	–
V10 (%)	0.37 (0.06–2.27)	0.29	–	–	0.44 (0.10–1.92)	0.28	–	–
V20 (%)	0.36 (0.02–5.27)	0.46	–	–	0.23 (0.02–2.27)	0.21	–	–
Mean (Gy)	0.98 (0.93–1.04)	0.48	–	–	0.97 (0.93–1.02)	0.29	–	–

CI = confidence interval; ENI = elective nodal irradiation; FEV1 = forced expiratory volume in 1 second; FEV1/FVC = forced expiratory volume % in 1 second; HR = hazard ratio; ILA-SF = subpleural fibrotic interstitial lung abnormalities; IMRT = intensity-modulated radiotherapy; KL-6 = Krebs von den Lungen-6; PD-L1 = programmed cell death-ligand 1; %VC = vital capacity percentage; Vx = percentage of lung volume receiving > x Gy.

The multivariate analysis revealed that ILA-SF was the only independent adverse factor for OS (HR: 3.07, 95% CI: 1.17–8.10, *p* = 0.02) and that it also tended to influence PFS (HR: 1.95, 95% CI: 0.91–4.14, *p* = 0.08). In contrast, younger age, adenocarcinoma, concurrent chemotherapy, and durvalumab treatment contributed to the prolongation of OS and/or PFS.

## Discussion

To the best of our knowledge, this is the first study to classify patients with NSCLC according to the Fleischner Society ILA subtypes and to demonstrate subtype-associated risk with respect to radiotherapy outcomes. In our analysis, ILAs were considered risk factors for grade ≥ 2 RP. Among radically treated patients, the ILA group showed significantly worse 2-year OS than the normal group. This was particularly true for the ILA-SF group, which also independently contributed to OS in the multivariate analysis.

To date, only a few studies have examined the relationship between ILAs and RP. A previous study retrospectively analysed the association between RP and original ILA scores in 145 patients with NSCLC.^9^ The ILA scores were rated as follows: 0, no interstitial lung change; 1, ILAs without honeycombing (ground-glass attenuation, fine reticular opacity, and microcysts); and 2, honeycombing. They concluded that abnormalities, with or without honeycombing, were predictors of RP. Several reports indicate that ILA is a risk factor for RP in patients with small-cell lung cancer.^15,16^ Conversely, another study concluded that ILAs did not independently contribute to RP.^10^ This discrepancy may be due to the ambiguous previous definition of ILAs. Moreover, previous studies have tended to confuse smoking-related centrilobular nodularity and pleuroparenchymal fibroelastosis with ILAs.^19,20^ These disease concepts are explicitly excluded from the novel definition of ILAs. Awareness of the improved definition of ILAs is essential for correct diagnosis, which should be made before radiotherapy to guide treatment strategy. Grade ≥ 2 RP occurred at a rate as high as 71 (41%) patients in this study. We believe that one of the important factors was the large number of patients with ILA included, 64 (37%), as a characteristic of the institution. If the presence of ILA had been accurately recognised in advance, countermeasures could have been taken.

The leading treatment-related factor that predicts RP is lung V20.^19,20^ V20 should be further reduced when radiotherapy is administered to patients with ILAs. Moreover, the use of IMRT should be considered along with techniques such as virtual lung block and end-inspiration irradiation.^21,22^ However, the ROC analysis in the ILA group showed low predictive accuracy for grade ≥ 2 RP using V20 (AUC = 0.59). This finding implies that reducing the lung dose in patients with ILAs may not necessarily preclude severe RP.

ILA subtype divisions demonstrated notable differences. The ILA-SF group had the highest cumulative incidence of grade ≥ 2 RP, corroborating previous studies that have shown ILA-SF to be 6.6 times more likely to progress than other subtypes.^23^ Contrarily, no difference was observed in the cumulative incidence of grade ≥ 2 RP between the ILA-NS and normal groups. The course of ILA-NS is usually non-progressive, suggesting a favourable prognosis.^21^ ILA-NS may not be a risk factor for RP; nonetheless, we emphasise that there were few patients with ILA-NS in this study; therefore, we cannot mention its clinical significance. Therefore, this subtype should be investigated in more eligible patients in the future.

No consensus exists that patients with ILAs have a poor prognosis after radiotherapy. However, studies have reported that the outcomes of surgery and chemotherapy for patients with lung cancer and ILAs are poor.^24,25^ In this study, all eight patients with grade 5 RP were in the ILA group, and seven of these patients were in the ILA-SF category. We believe that the severity of RP worsened the prognosis of the ILA group, particularly that of patients with the ILA-SF subtype. The advent of durvalumab has greatly improved the prognosis of NSCLC; however, it is difficult to administer in the long term, particularly in patients with ILASF. Death sometimes results from severe RP rather than cancer; therefore, the indication for radiotherapy should be carefully considered.

Our study had some limitations, particularly its retrospective and single-institution design. Additionally, other factors, such as emphysema, chronic obstructive pulmonary disease, and biomarkers (e.g., transforming growth factor-beta), may contribute to RP.^7^ These factors, which we did not consider in this study, may be confounders. Furthermore, many of our patients were elderly and frail and were treated with radiotherapy alone. We also included several cases from the period before the emergence of durvalumab. These differ from modern standard treatments and may have implications for survival analysis. Retrospective studies on cases with durvalumab have been reported recently^26^, and high-quality future prospective trials are warranted

## Conclusions

The novel definition of ILAs and ILA subtypes may be important in determining risk for RP following radiotherapy in patients with NSCLC. RP symptoms can be severe, especially in patients with ILA-SF, and may worsen prognosis. This study suggests that recognising the presence of and categorising ILAs before treatment is vital and useful in clinical decision-making.
